# Editorial

**Published:** 1867-08

**Authors:** 


### Editorial.

CHICAGO MEDICAL SOCIETY.—The meetings of this society, which have been comparatively well attended during the past year, are now being held once in two weeks. During the past six months, while the meetings have been held once a week, many very interesting specimens of pathological anatomy have been presented, chiefly by those members connected with the public hospitals. The relation of cases, and the discussion of questions of direct practical importance have occupied the chief attention of the Society.

At the last meeting, Dr. EARLE read a report on the treatment of angular curvature of the spine, embracing several cases, with photographic representations of different stages of improvement while under treatment, and an exhibition of the mechanical appliances resorted to with great benefit. The mechanical treatment pursued by Dr. EARLE is essentially the same as that recommended in the small works of Drs. TAYLOR, of New York, and LEE, of Philadelphia.

Dr. DANFORTH, presented a tumor which had been removed from beneath the chin. It had been over seven years in growing from the size of a small pea to that of a large hickory-nut. It consisted of a dense inorganic formation, apparently of lime, enclosed in a cyst or membrane, with a small quantity of serous fluid. Before removal, it was freely moveable under the skin, and had never occasioned pain or soreness.

Dr. Lackey reported a case of gunshot wound, in which the ball wounded the median nerve of the arm. After the wound had cicatrized, the hand remained paralyzed and extremely painful, indicating, as was supposed, pressure on the nervous cord at the place of injury. An operation was resorted to, but instead of finding anything pressing on the nerve, close search disclosed a small piece of the lead from the ball imbedded in the nerve. This was removed, and the patient recovered both from the pain and paralysis. Dr. L. thought many of the cases of pain and paralysis following wounded nerves might be the result of the presence of a foreign substance instead of pressure.

Dr. Lyman related a case seen by him in the Massachusetts General Hospital, in which an elderly woman had the fore-arm amputated below the elbow, for the removal of a paralyzed and terribly painful hand, with which she had been affected four or five years continuously. It had been produced by part of a needle retained in the palm of the hand. Dissection of the hand disclosed half an ordinary sewing needle imbedded beneath the palmer fascia, in such position that each end was in contact with a nerve.

Dr. Brink read a short paper on the etiology of goitre, suggesting that iodine taken in the various articles of food, etc., was the special cause of the disease.

At a more recent meeting of the Society, Dr. H Wanzer reported an interesting case of concussion of the brain from a blow, followed by the discharge of a large quantity of blood, and protracted paralysis of the nerves supplying the muscles and coats of the eyes. This led to an interesting discussion, which was participated in by Drs. Durham, Hildreth, Bogue, and Wanzer. Dr. Hildreth, who saw the case some time after the injury, said it presented a well-marked case of anæsthesia of the cornea, accompanied by contraction of the pupil, which was relieved by atropia. Dr. Bogue, who attended the patient after he was removed to the County Hospital, thought there had been neither fracture of the skull nor effusion of blood forming a clot on the brain, but that the concussion had seriously disturbed the structure of the brain at the origin of the 5th, 6th, and 7th pairs of nerves. There was at no time decided paralysis of either upper or lower extremities. The patient recovered a fair degree of general health, but the recovery from paralysis of the nerves of the eye and face is incomplete.

Dr. E. L. Holmes reported a case of amaurosis connected with albuminuria, in which the ophthalmoscope revealed the yellow circular spots and dark granules of extravasated blood, characteristic of such cases. In this case, the rings that are usually yellow were bright red.

Dr. J. S. Hildreth mentioned a case presenting similar appearances, connected with albuminous urine and pregnancy. He thought the same condition of the retina had also been observed in some cases of diabetes.

Reports in relation to the sanitary condition of the city and the prevalence of diseases, during the months of April, May, and June, were presented by Drs. R. G. Bogue and N. S. Davis. These elicited remarks from Drs. Wickersham, Bevan, and Fitch. One of these reports may be found in the present number of the Examiner.

---

### GEORGE K. AMERMAN, M.D.

At a meeting of the Chicago Medical Society, held at the Court House, June 28, Dr. J. P. Ross announced to the Society that a brother member, Dr. George K. Amerman, had departed this life, at Marcellus, N. Y., June 20, 1867. For nearly two years his health had been failing, and in April he had abandoned the practice of his profession, going home to die of consumption. Dr. Ross moved the appointment of a committee to prepare a series of resolutions appropriate to this sorrowful occasion. The motion having prevailed, the following gentlemen were appointed by the Chairman: Drs. Ross, Holmes, Bevan, Heydock, and Marguerat. They reported as follows:

Having been informed of the death of their associate, George K. Amerman, M.D., the members of the Chicago Medical Society desire to testify for the deceased their respect, and their feelings of personal loss by these resolutions:

*First.* That in this affliction we lament the death of one who, long identified with our community, though young in years, was old in professional renown. A man whose life was a career of brilliant success, a Christian in deed, as well as in name, at the height of his reputation, he has now received the crown of immortality.

*Second.* That to the members of our profession we earnestly commend the example afforded by the life of our departed associate.

*Third.* That to the surviving relatives of our beloved friend we tender this expression of our sympathy in view of their bereavement, ever desiring with them to bow in humble acknowledgment of the almighty power of that God in whose hand are the issues of life and of death.

*Fourth.* That a copy of these resolutions be furnished to the relatives of the deceased, and to the medical journals and daily newspapers of the city.

The report of the Committee was accepted.

Dr. Ross then read a technical history of the last illness of the departed brother.

Dr. J. R. Gore, who had known Dr. AMERMAN from infancy, being invited to address the Society, proceeded to relate the following interesting facts concerning his friend:

At the time of his death Dr. Amerman was in his thirty-fifth year. The son of a rural farmer in Central New York, his boyhood was distinguished by fondness for study, and by disinclination to the drudgery of farm work. He was obedient and kind, but *good for nothing* on the farm. He early manifested a passion for teaching, and at the age of sixteen his only ambition was to become a school-teacher. His father, however, proposed that he should study medicine. To this he consented. Placed under the guidance of Dr. Gore, then a practicing physician in Owasco, N. Y., he pledged himself to pursue the study of medicine, subject to the direction of his preceptor, for five years before attempting to commence its practice. He accordingly attended three courses of lectures in the University Medical College of New York, graduating with honor in the spring of 1854. Still acting under the guidance of his preceptor, he secured a position for one year on the resident medical staff of Bellevue Hospital. The next year he became a member of the resident surgical staff of the same hospital. There, in the enjoyment of the friendship and confidence of the most distinguished physicians and surgeons of New York City, Dr. Amerman completed the fifth year of his course of study. At once received into partnership by his former preceptor, and removing to Chicago, he commenced the practice of his profession in 1856. He was appointed Surgeon to the Illinois Central Railroad, an office which he filled with great credit until the time of his death. He was also one of the Surgeons to the City Hospital, until that institution was closed during the war, and was the leading spirit in the surgical staff of the County Hospital from the time of its renovation until failing health compelled him to surrender his large and lucrative practice.

After listening to the remarks of different gentlemen who united in eulogizing the memory of the deceased, the resolutions were adopted, and the Society adjourned.

J. P. ROSS, M.D., President.

HENRY M. LYMAN, M.D., Secretary.

ACTION OF THE HOSPITAL BOARD.

At a meeting of the Medical Board of the County Hospital, Chicago, to take action on the death of Dr. GEORGE K. AMERMAN, the following resolutions were adopted:

*Resolved*, That the members of this Board have heard, with feelings of the deepest sorrow, the death of their colleague, Dr. Amerman, and desire to put on record their high appreciation of him as a gentleman and a surgeon.

*Resolved*, That in his death the staff of this Hospital has lost a most efficient member, and one most intimately identified with its best interests; that the profession at large has lost one of its brightest ornaments, and our community a most valuable cltizen.

*Resolved*, That we believe his death was the result of too arduous devotion to his chosen calling, and that he has added another to that long list of glorious martyrs who have given up their lives in the cause of suffering humanity.

*Resolved*, That a copy of these resolutions be sent to his friends at the East, and to the public journals.

| | |
|---|---|
| R. C. HAMILL, M.D., | H. A. JOHNSON, M.D., |
| J. P. ROSS, M.D., | R. G. BOGUE, M.D., |
| H. W. JONES, M.D., | CHARLES G. SMITH, M.D., |
| THOS. BEVAN, M.D., | H. M. LYMAN, M.D., |
| J. R. GORE, M.D., | J. S. HILDRETH, M.D. |

---

NEW COLLEGE BUILDING.—On the 28th of May, the corner-stone of the new building for Rush Medical College, was laid with the usual ceremonies of the Masonic Order. An address was delivered by Prof. J. A. ALLEN, and a very good audience was in attendance. The new building is to be on the same lot, and in direct communication with the old one. The recently appointed Professor of Surgery, Dr. MOSES GUNN, formerly of Detroit, has also taken up his permanent residence in this city.

---

MEDICAL CONVENTION IN WISCONSIN.—We learn that a convention, embracing a large number of the leading physicians of Wisconsin, was held in Janesville, on the 23d and 24th of July. The object was to excite a more active interest in the social organization of the profession in that State. It is a move in the right direction, and we hope to see it followed up vigorously.

### Mortality Report for the Month of June:—

### CAUSES OF DEATH.

Below is given the mortality report for the month of June, just ended.

From this report and a comparison of it with the report for the same month of last year, it appears that the city is at the present time in a healthy condition. The number of deaths last year in June was 319—this year 283—decrease, 36.

| | | | |
|---|---|---|---|
| Accidents, | 8 | Fever, Typhoid, | 15 |
| Asthma, | 1 | Fever, Child-bed, | 7 |
| Abortion, | 1 | Fever, Ship, | 3 |
| Apoplexy, | 2 | Fever, not stated, | 1 |
| Acute gastretis, | 2 | Hydrocephalus, | 1 |
| Burned, | 4 | Inflammation of Bowels, | 5 |
| Cancer, | 3 | Inflammation of Brain, | 4 |
| Cold, | 1 | Inflammation of Lungs, | 2 |
| Croup, | 2 | Killed, | 5 |
| Consumption, | 35 | Marasmus, | 3 |
| Convulsions, | 31 | Measles, | 8 |
| Congestion of brain | 2 | Osteomyletus, | 1 |
| Congestion of Lungs, | 1 | Old Age, | 9 |
| Congestive Chills, | 1 | Poisoning, | 1 |
| Drowned, | 12 | Paralysis, | 3 |
| Decline, | 1 | Pneumonia, | 1 |
| Diarrhœa, | 2 | Palsy, | 1 |
| Diphtheria, | 1 | Rheumatism, | 1 |
| Dropsy, | 8 | Suicide, | 1 |
| Disease of Brain, | 2 | Spinal, | 4 |
| Disease of Heart, | 3 | Stillborn, | 13 |
| Disease of Lungs, | 1 | Sunstroke, | 2 |
| Disease of Kidneys, | 1 | Small-Pox, | 4 |
| Disease not stated, | 1 | Summer Complaint, | 9 |
| Dysentery, | 1 | Teething, | 4 |
| Erysipelas, | 4 | Whooping-Cough, | 4 |
| Fever, Bilious, | 1 | Unknown, | 26 |
| Fever, Brain, | 5 | | |
| Fever, Scarlet, | 8 | Total, | 283 |

| | |
|---|---|
| Total number last year for the month of June, | 319 |
| Total number during the month of June, | 283 |
| Total number during the month of May, | 241 |
| Increase, | 42 |

### DIVISIONS OF THE CITY.

| | | | | | | | |
|---|---|---|---|---|---|---|---|
| North, | 64 | South, | 91 | West, | 124 | Total, | 283 |
| Unknown, | 4 | | | | | | |

Ages of the Deceased. —Under 5 years, 139; over 5 and under 10 years, 10; over 10 and under 20, 19; over 20 and under 30, 29; over 30 and under 40, 39; over 40 and under 50, 14; over 50 and under 60, 9; over 60 and under 70, 6; over 70 and under 80, 5; over 80 and under 90, 4; unknown, 9. Total, 283.

### NATIVITIES.

| | | | | | |
|---|---|---|---|---|---|
| Chicago, | 143 | Germany, | 32 | Sweden, | 7 |
| United States, | 23 | Holland, | 2 | Switzerland, | 1 |
| Canada, | 5 | Ireland, | 35 | Scotland, | 2 |
| Bohemia, | 3 | Italy, | 1 | Unknown, | 16 |
| England, | 4 | Norway, | 10 | On Sea, | 1 |

Total, 283

Surgeon J. H. Baxter of U.S. Volunteers.—This active and hard working member of the Medical Staff of the Army, has recently been appointed Assistant-Medical-Purveyor, with the rank of Lieutenant-Colonel in the U.S.A. The appointment has been confirmed by the Senate.

---

Chlorate of Potassa in Pneumonia.—Dr. J. C. Morley, of Mississippi, recommends strongly the use of chlorate of potassa in doses of from five to twenty grains, repeated every one or two hours, as a remedy in the treatment of pneumonia. We have used the remedy in the treatment of the more severe attacks of pneumonia for eight or ten years past, and with decided benefit.

---

Money Receipts from June 26th to July 26th.—J. F. Kelsey, Hortonville, Wis., $3; F. R. Payne, Marshall, Ill., 3; F. H. Jennings, Marshall, Ill., 3; Nathan Spencer, Marshall, Ill., 3; Daniel Gard, Martinsville, Ill., 3; Jesse Compstock, Westfield, Ill., 3; J. C. Price, Westfield, Ill., 3; W. S. Goodell, Westfield, Ill., 3; J. D. Mitchell, Darwin, Ill., 3; J. A. Patton, Livingston, Ill., 1 50; Henry Biroth, Chicago, 1 50; John O'Reilly, New York, 3; A. E. Van Deventer, Oswego, Ill., 3; John D. Cope, Fairfield, Ill., 3.

